# Health traditions of *Sikkim* Himalaya

**DOI:** 10.4103/0975-9476.72617

**Published:** 2010

**Authors:** Ashok Kumar Panda, Sangram Misra

**Affiliations:** *Ayurveda Regional Research Institute, Tadong, Gangtok, Sikkim - 737 102, India*; 1 *Department of Basic Principle, Rajiv Gandhi Medical College and Hospital, Durg, Chhatisgarh, India*

**Keywords:** Ayurveda, folk healers, medicinal plant, *tibetan* medicine

## Abstract

Ancient medical systems are still prevalent in *Sikkim*, popularly nurtured by *Buddhist* groups using the traditional *Tibetan* pharmacopoeia overlapping with Ayurvedic medicine. Traditional medical practices and their associated cultural values are based round *Sikkim*’s three major communities, *Lepcha, Bhutia* and *Nepalis*. In this study, a semi-structured questionnaire was prepared for folk healers covering age and sex, educational qualification, source of knowledge, types of practices, experience and generation of practice, and transformation of knowledge. These were administered to forty-eight folk healers identified in different parts of *Sikkim*.

490 medicinal plants find their habitats in *Sikkim* because of its large variations in altitude and climate. For 31 commonly used by these folk healers, we present botanical name, family, local name, distribution, and parts used, together with their therapeutic uses, mostly Rheumatoid arthritis, Gout, Gonorrhea, Fever, Viral flu, asthma, Cough and Cold, indigestion, Jaundice etc. A case treated by a folk healer is also recounted. This study indicates that, in the studied area, *Sikkim’s* health traditions and folk practices are declining due to shifts in socio-economic patterns, and unwillingness of the younger generation to adopt folk healing as a profession.

## INTRODUCTION

The three ethnic groups of *Sikkim*, the *Lepcha*, *Bhutia*, and *Nepalis*, have long practiced their traditional systems of medicine and have a strong belief in herbs. 490 medicinal plants find their habitat in *Sikkim* due to its large variations in altitude and climate.[1] *A*ncient medical systems abound in *Sikkim*, and are still popular, nurtured by *Buddhist* groups for their traditional *Tibetan* Pharmacopoeia.[2] The tribals of *Sikkim* have immense faith in herbal medicine based on trial and error experience gained continuously from generation to generation. The various traditional healers across the globe have diverse beliefs and practices, but the common aim of all is to cure ailments and maintain human health. Any medical system operates in society according to the prevailing environment of the region and cultural manifestations operating within it.[3] In *Sikkim Himalaya*, geographical factors have not only contributed to this, but also prevented close contact with other developed indigenous systems of medicine. Human societies living in high-altitude areas remain isolated due to poor accessibility and harsh climate. Their geographical conditions stimulate them to develop a unique health culture. In *Sikkim*, this is a mixture of *Lepcha, Bhutia*, and *Nepali* practices for the prevention of disease, promotion of health, and treatment of disease. These unique practices are undocumented and passed on from one generation to next by word of mouth.[4] Studies are available concerning demographic and practice patterns of folk medicine in other developing counties. Such kinds of serious work are rarely available in India, although we have a strong knowledge base, strong belief, and acceptance.[5] So the present study aimed to document the various plants used by *Sikkim*’s folk healers, practice patterns, demographics, and their socioeconomic condition with the future prospects of their tradition.

## MATERIAL AND METHODS

### Study area

The present study is mainly focused on health tradition, various plants used by the folk healers, and their socioeconomic status present in three ethnic groups (*Lepha, Bhutia*, and *Nepali* communities). The study area encompassed four of *Sikkim*’s districts.

### Study methods

Different trips were arranged to different places in *Sikkim* to identify folk healers. The study was conducted in collaboration with the State Medicinal Plant Board to identify the Folk healers. The healers were identified or selected as per their reputation as narrated by local people. We conducted one training workshop to educate the healers about their strengths and weaknesses, and to give fundamentals on vital sign. We interacted with the healers and collected personal data together with information about their feelings. Raw materials used for curing different ailments, and folk healers’ beliefs concerning them were collected and complied during field surveys and workshops over the period January 2007 to December 2008. A semistructured questionnaire was prepared for folk healers in whch age and sex, educational qualification, source of knowledge, types of practices, experience and generation of practice, and transformation of knowledge were studied in 48 identified folk healers in different areas of *Sikkim*.

## RESULT AND DISCUSSION

### Brief history of health culture in *Sikkim*

*Sikkim* is known to the *Lepchas* as “*Nye-mael-liang*” meaning *paradise*. It is also called “Ren-Jong” meaning the *land of ancestors*. The present name *Sikkim* is of Nepali origin and derived from *Sukhim* meaning new house or new place. Another derivation from the Sanskrit word *Sikhim* suggests the meaning *mountain country. Lepchas* are an indigenous tribe not only in *Sikkim* but also in north *Bengal*. Besides them, the *Bhutia* (from Tibet) and *Nepalis* (from Nepal) are also inhabitants of *Sikkim*.

Historically, *Sikkim* did not have any strong centralized political power before the 17^th^ century. It is also accepted that the Lepchas were the autochthonous people of the state who had long before migrated there from South East Asia. The contact between Lepchas and Bhutias is believed to have begun around 1275 AD. After the coronation of the first Chogyal (king) in 1642, a treaty was signed between them.[6]

Culture is the foundation of civilization, and a social strength, which is enriched by the transforming power of knowledge and experience. The health culture of *Sikkim* is a composite heritage of practices of medicine in different rituals and other measures of prevention and cure.[7]

These three major *Sikkim* communities practice their systems of traditional medicine based on their cultural values as follows:

#### Lepcha medical practices

Lepchas constitute about 13% of the total population of the state they inhabit in the Dzongue reserve of North *Sikkim* district. The concept of health and illness among the Lepchas is entirely guided by belief in the supernatural. The Lepchas mainly follow the *Mahayana* sect of Buddhism. They have their own script, and distinct costume, language, and culture. Uses of medicinal plants are described in the Lepcha epics called Namthar, Tengyur, and Domang. They acknowledge certain semidivine beings or guardian spirits known as “Lungzee,” who are not gods but worthy of respect like a huge tree, a cluster of trees, grass, a tarn, a cave or a special hillock, and other natural objects. If they are ignored or any disrespect is shown to them by defiling or polluting, by answering nature’s call etc., it may invite suffering to the village or the particular individual; they may suffer from serious sickness or even die.

According to the Lepchas, the world is governed by good spirits – and evil spirits (Mung). All natural calamities such as bad harvests, draught, hailstorm, and other misfortunes are believed to be the actions of evil spirits, i.e., Mung. On the other hand, good health and vitality, good harvest, and prosperity are attributed to actions of good spirits.[8]

Since the Lepchas are basically animist, traditionally only the *Bongthings* (male Lepcha priests) and *Muns* (the female Lepcha priestess) are called during sickness and for cultural and funeral ceremonies. Such is the influence of the mun/bong things, that even after the introduction of Buddhism, the lamas performed their pujas in close association with them. The Mun, however, perform rituals connected with supernatural forces in which the lamas have no role.

*Pougorip/Totola* (Oroxylum indicum)[9] is a medicinal plant used in Ayurveda as an ingredient in *Dashamula*. It also plays an important role in the *Lepcha* culture. The Lepchas believe that it is not even touched by the bees, signifying the purity and chastity of a virgin girl, and it is used as liver tonic and antidiabetic medicine. The fruit of the plant is shaped like a huge sword. The seeds from inside the fruit are flagellated like paper silk, and are used in any auspicious ceremony similarly to the use of haldi/turmeric in Hindu culture. Chi (millet beer) plays a very important role in Lepcha culture and used to drink to good health.[10]

#### Bhutia medicinal practices

The Bhutias place great emphasis on coercive rites of to exorcise and destroy demons. Like the Lepchas, the execution of religion is in the hands of trained specialists called *pau, neyjum*, and lamas, *paus* being male and *neyjums* female. During the process of curing, a pau enters a trance state, to communicate with the spirits and discover why they have afflicted the patient with illness. Another approach to diagnosis is by divination with the help of a plate full of rice. The pau shakes the plate until the symbol of the evil spirit appears in the rice. The *pau* performs *Phuphi* by offering money, eggs, and clothes which have been circulated thrice over the patient’s head to the malignant spirit.[11] These things are thrown out and only the clothes are brought back. It is believed that patients will be cured within three days of the ritual.

All *Sikkim* people’s settlements are adorned with prayer flags, or *Dacho*, which are said to carry good fortune to the individual in every direction. These flags are of four types – the Lung-ta which is square in form, and contains a horse with mystic figure at the center. It is hung on the ridges of the house and in the vicinity of settlements; the chonpen, long, narrow and rectangular in shape, is tied to twigs of trees or to bridges or to bamboo flag posts; the *Gyal-tsen dse-mo*, which is like *lung-ta*, but contains a larger holy text; and the great luck charm, which is pasted on the walls of the house or folded up and worn around the neck as a charm for good fortune. Luck flags are flown only after performing certain specific *lamaic* worship. Waddell states that most of the *lamaic* worship is derived from demonolatry, “A few of the most intelligent *Lamas* become *Tsi-pa lamas* who are astrologers. As in the rest of South Asia, all the laity understand that astrological choice of time is absolutely essential for each of the three great epochs of life, viz., birth, marriage and death; and also at the beginning of each year to have a forecast of the year’s ill-fortune, such as health problems, and to have appropriate remedies drawn up.”

#### Nepali medical practices

*Nepalis* believe that supernatural forces are involved in the creation of illness. *Dami* and *Jhakries* are performed during the *puja* for physical and mental diseases and *Phedangba* in particular for the *Limboo* community. Folk uses of herbs such as *Oroxylum indicum* (hypertension), *Fraxiknus floribunda* (gout), *Panax pseudoginseng* for longevity, *Ephedra gerardiana* for asthma, *Elshcolzia blanda* and *Mahonia nepalensis*[12] in eye-trouble and eczema, and of *Urtica parviflora* (young inflorescence) as a clearing and invigorating agent after child-birth by local women folk, are of great value. Rhizome of Budo-Vokati (*Stible rivlaris*) is considered to be good for lumbago.[13] It is crushed and taken as decoction after boiling in water or chewed like betel nut for relief of body ache. Flowers of *Pandanus nepalensis, said to be aphrodisiac and induce sleep, are* found in *Sikkim* up to 1752 m altitude and worn by girls in their hair to win their lovers. The plant’s roots taken with milk are said to prevent abortion; the flowers are said to remove headache and weakness, and their seeds to cure broken hearts.[14]

The healing practices of these three ethnic groups are a mixture of personalistic and naturalistic theories of illness. According to personalistic theories, illness may be linked to transgressions of a moral or spiritual nature. They may involve inappropriate behavior, violation of social norm, or breaches of religious taboos on the part the patient. Naturalistic theories view illness as a disharmony between the person and the environment. Perception of illness is highly culture related.[15]

### Uses of medicinal plants in *Sikkim*

Medicinal plants used by the different folk healers are presented in alphabetic manner with Latin name, distribution, parts used, and the specific disease for which it was used [Table 1].

**Table 1:** Medicinal plants with their uses by folk healers of *Sikkim*

Botanical name	Family	Local name	Distribution	Parts used	Medicinal/ Other uses
Aconitum bisma	Ranunculaceae	Bikhma	Alpine zone	Tubers, roots	Tuber is used in food poisoning, asthma, cough and bronchitis
Aeschynanthus sikimensis	Gesneriaceae	Baklay patay	5000 to 7000 ft	Rhizome	Decoction of root is used in fever and throat pain
*Aesculus indicus*	Sapindaceae	Pangra	Lower hill forest	Fruits	Seed oil used in rheumatism and mumps
*Aesandra butyracea*	Sapotaceae	Chewri	Middle hill forest	Fruits	Used in rheumatism
*Allium wallichii*	Alliaceae	Bana Lasuna	Sub alpine region	Leaves	Viral flue and used in high altitude sickness
*Artemisia vulgaris*	Compositae	Titepati	2000–5000 ft	Leaf decoction	Leaf decoction used on cuts and bruises to stop bleeding mostly in nose bleeding and measles and fever.
*Bergenia ciliata*	Saxifragaceae	Pakhan bhed	Upper hill forest	Rhizomes roots	Used in fever and applied to boils; rhizome in white discharge
*Betula utilis*	Betulaceae	Bhoj patra	Upper hill forest	Bark	Used to heal up wounds from bone fracture
*Bischofia javanica*	Euphorbiaceae	Kainjal	Middle hill forest	Leaves, bark	Fruits are used in making wine; stem bark is used for irregular menstruation and pain
*Brugmansia suaveolens*	Solanaceae	Kolo dhaturo	Middle hill forest	Leaves	Applied to cure swellings, sprain and rheumatism
*Buddleja asiatica*	Buddlejaceae	Bhinsen pati	Lower hill forest	Leaves, flowers, stem	Used for skin problems and as abortificant
*Cordyceps sinensis*	Clavicipitaceae	Yarcha gombuk	Alpine	Whole plant	Rejuvenates liver, heart, and retards aging processes in the immune system
*Daphne bholua*	Thymelaeaceae	Kagatey	Upper hill forest	Bark and Root	Bark decoction given to treat fever; root bark used for intestinal worms
*Dioscorea deltoidea*	Dioscoreaceae	Kurkurtarul	Lower hill forest	Bark and tuber	Tuber used in rheumatoid arthritis, asthma, and fever
*Ephendra sikkimensis*	Ephedraceae	Somlata	Lower hill forest	Whole plant	Plant raises blood pressure and used to relieve high fever, gout, and arthritis.
*Eupatorium cannabium*	Asteraceae	Banmara	Lower hill forest	Leaves/stem	Leaf and stem extract used on cuts and bruises to stop bleeding and infection
*Fraxinus floribunda*	Oleaceae	Lakuri	Middle hill forest	bark	Bark boiled and applied for gout, sprain, and used in fracture
*Heracleum wallichi*	Apiaceae	Chimphing	5000 to 7000 ft	Fruits and root	Fruits used orally during influenza, root as aphrodisiac
*Lindera neesiana*	Lauraceae	Timbur	Temperate Himalayas	Bark and fruits	Flower used for excessive seminal discharge in dream; fruits are used to induce vomiting
*Marsdenia roylei*	Asclepiadaceae	Bahuni Lahara	Lower hill forest up to 5000 ft	Roots and leaves	Cooling effect in gonorrhea
*Nardostachys jatamansi*	Valerianaceae	Jatamansi	Above 7000 to 9000 ft	Root	Root used for hair loss, in epilepsy and hysteria
*Orchis sp. latifolia*	Orchidaceae	Panchamala	Above 8000 ft	Root tuber	Root tubers highly nutritious, used as aphrodisiac
*Oxalis corniculata*	Oxalidaceae	Chari amilo	Lower hill forest	Whole plant root	Leaf juice taken to cure dysentery and fever anemia and tympanitis for appetite digestion
*Panax pseudoginseng*	Araliaceae	Mangan	Above 8000 ft	roots	Root taken to reduce fever, indigestion and vomiting also used as tonic
*Picrorhiza Kurrooa*	Scrophulariaceae	Kutki	Above 7000 ft	Roots	Used as laxative, brain tonic, emetic, good in paralysis, Jaundice
*Podophylum hexandrum*	Berberidaceae	Papari	Alpine hills	Whole plant	Cures septic wounds and diarrhea
*Rubia monjita*	Rubiaceae	manghito	Upper hill forest	Stem root	Root tonic cures skin diseases, stem used for scorpion sting
*Saussurea gossypiphora*	Asteraceae	Kapis ful	Lower hill forest	Plant/root	Plant paste used for cuts and bruises; root paste used to cure cough, asthma, fever, and dysentery; inflorence used for sexual dysfunction
*Stephania glabra*	Menispermaceae	Taubarkey	Lower hill forest	Root bulb	Powder used in diabetes tuberculosis, asthma, fever
*Swerita chiraita*	Gentianaceae	Chiraita	Upper hill forest	plant	Plant juice used to cure malaria fever
*Taxus bacata*	Taxaceae	Dhengresalla	Above 8000 ft	Leaf/ bark	Leaf extracts used in breast and throat cancer

In this study, data were collected on a total of 44 medicinal plants, commonly used by 48 folk healers. These plants are presented by botanical name, family, local name, and distribution with medicinal uses. Most of the plants are used for the following complaints rheumatoid arthritis, gout, gonorrhea, fever, viral flu, asthma, cough and cold, indigestion, etc. [Table 1]. A total of 48 folk healers were identified in four districts of *Sikkim* and with the largest number 18(37.5%) folk healers in East *Sikkim* district. [Table 2]. Their age and sex, educational qualifications, sources of knowledge, types of practices, experience and generation of practice, and transformation of knowledge are as follows.

**Table 2:** District-wise habitat of identified 48 folk healers of *Sikkim*

District name	Number of folk healers	Percentage
East Sikkim	18	37.5
West Sikkim	17	35.41
South Sikkim	06	12.5
North Sikkim	07	14.58
Total	48

**Graph 1:**
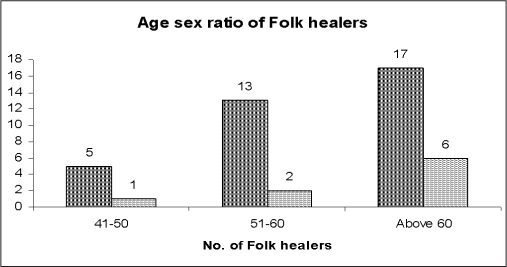
Age and sex distribution of 48 identified folk healers of *Sikkim*

- Only 4 (8.32%) of folk healers were young i.e. in the age range (20–40) years, and 17 (47.92%) were over the age of 60. Therefore it is mandatory to acquire the knowledge from the elderly; otherwise folk healing tradition will vanish from *Sikkim*. The study shows that most folk healers 39 (81.25%) were male while 9 (18.75%) were female [Graph 1]. About half 25 (52.08%) were illiterate and only 5 (10.41%) had education beyond matriculation. [Table 3]
- Twenty-seven (56.25%) healers acquired their knowledge from their parents, and 11 (22.91%) acquired it from their Guru. Only 4 (8.34%) learned herbal medicine by reading books and manuscripts [Table 4]. Bone setting is the dominant traditional used by 23 (47.91%) folk healers is the dominant traditional practice. Only 2 practice veterinary medicine; only 1 treats snake bite [Table 5].
- Most 34 (70.84%) of the folk healers belong to the Nepali community and only 4 folk healers belong to the Lepcha community [Table 6]. An attempt was made to understand the relevance of particular indigenous systems of medicine to practice among the folk healers of *Sikkim*. It was found that 19 (39.58%) of the Nepali folk healers practice in accordance with Ayurvedic principles of treatment, and 12 (25.00%) practice Tibetian medicine. No one is using the Siddha, Unani, or Yogic systems of indigenous practice. The majority of (68.75%) folk healers are practicing their tradition as third generation [Tables 7 and 8].

**Table 3:** Educational background of 48 identified folk healers

Education	Number of folk healers	Percentage
Illiterate	25	52.08
Up to class 5	10	20.84
Below matriculation	8	16.67
Above matriculation	5	10.41
Total	48	

**Table 4:** Sources of knowledge in 48 identified folk healers of *Sikkim*

Sources of Knowledge	Number of folk healers	Percentage
Traditional	27	56.25
Guru (Folk healing Teacher)	11	22.91
Own experiences	5	10.42
Books/Manuscript	4	8.34
Dreams	1	2.08
Total	48	

**Table 5:** Types of practices in 48 identified folk healers of *Sikkim*

Types of Practices	Number of folk healers	Percentage
Bone setting	23	47.91
General treatment	16	33.34
Vetenary medicine	2	4.17
Birth attendant	6	12.5
Poisoning treatment	1	2.08
Total	48	

**Table 6:** Types of community in 48 identified folk healers of *Sikkim*

Types of Community	Number of folk healers	Percentage
Lepcha community	04	8.33
Bhutia community	10	20.84
Nepali community	34	70.84
Total	48	

**Table 7:** Relevance of practice with indigenous medicine

Types of indigenous medicine	Number of folk healers	Percentage
Tibetan medicine	12	25
Ayurvedic medicines	19	39.58
Siddha/ Unani	Nill	-
No relation	17	35.42
Total	48	

**Table 8:** Generation of practice in 48 traditional folk healers of *Sikkim*

Generation of Practice	Number of folk healers	Percentage
1st	04	8.34
2nd	11	22.91
3rd	33	68.75
Total	48	

**Table 9:** Economic standard of 48 traditional folk healers of *Sikkim*

Average monthly income in Rupees	Number of folk healers	Percentage
1,000 to 3,000	23	47.91
3, 001 to 6,000	12	25
6,001 to 9,000	9	18.75
Above 9,000	4	8.34
Total	48	

**Graph 2:**
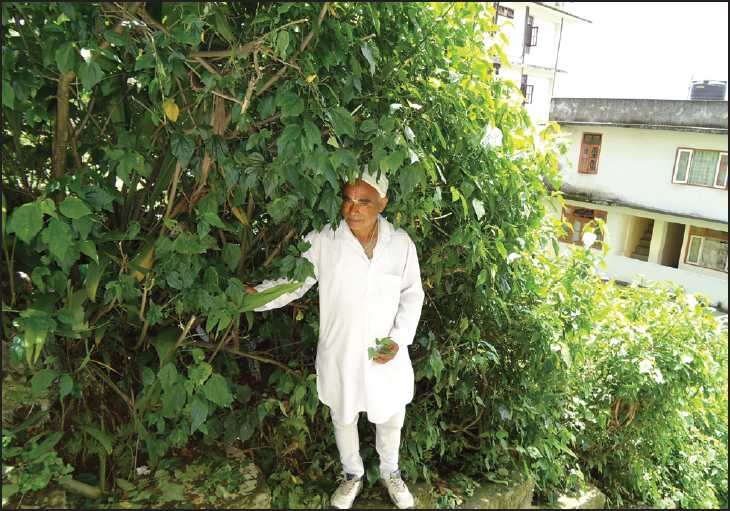
Status of transformation of knowledge of 48 Folk healers to maintain the tradition in *Sikkim*

The socioeconomic standards of *Sikkim* folk healers were also studied. It was found that most folk healers (48%) have a monthly income in the range Rs.1000–3000, while only 8% folk healers are earn over Rs. 9000 per month [Table 9]. Most importantly, 80% of the folk healers were ready to find alternative means of earning and wanted to leave their tradition. Also they are not happy with their profession. An attempt was made to know the ways of transformation of existing knowledge in the studied population. It was found that 28(58.33%) folk healers had not transferred their knowledge to anybody, even after the age of 50, 15(31.25%) folk healers had transferred their knowledge to their sons and daughters.[Graph-2]

The health traditions of *Sikkim* are linked with the ancient philosophical systems that make connections between the cosmic and terrestrial, between the outer and inner environment, and between the external and internal body. The people of *Sikkim* access folk medicine easily from the surroundings for little or no cost, and it is considered effective as well as acceptable as a method of treatment. It would be difficult to change the faith of elderly people in *Sikkim* in traditional medicine even if allopathic drugs were available. They are scared to use modern medicine as allopathic medicines are strong and chemical-based, need doctor’s prescription, and are not free of side effects. Health professionals need to understand that what patients believe about their illness and which methods of cure they consider effective and acceptable are as culture related as their perception of illness.

### Case study of folk healing practice

**Figure 1:**
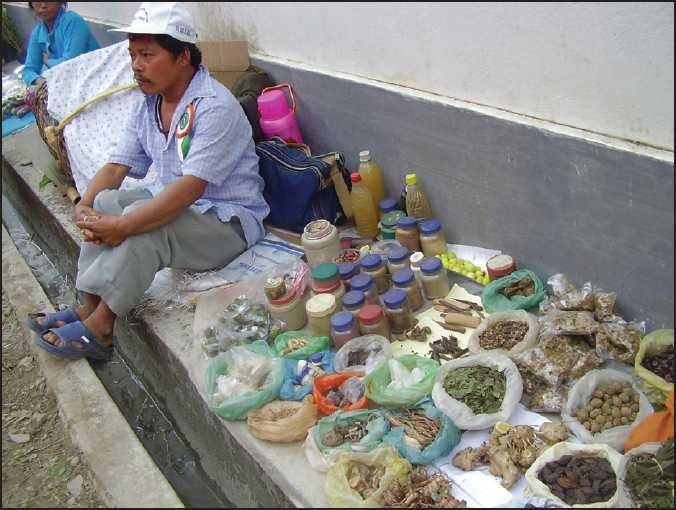
Traditional folk healer of Chengay Lakha, East Sikkim.

Mr Chintamoni Dabani [Figure 1] of Chengay Lakha, East *Sikkim*, is a traditional folk healer aged about 60 years; he has been successfully practicing herbal medicine for 40 years having acquired his knowledge from his father. He treats ailments such as fever, jaundice, gastritis, wounds, burn, female disorders, and infertility.

**Figure 2:**
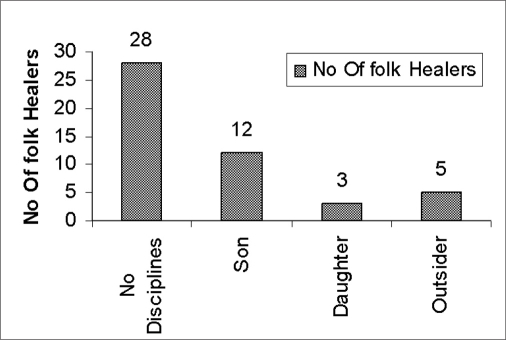
Folk healer of *Sikkim*

An example of his method of treating fever is as follows: a patient came to his house having been suffering from fever with headache for 2 days. He checked the patient’s pulse and advised him to take decoction of Swertia chirayata stem and leaves 3–4 times daily for 3 days. The patient returned to normal after 3 days without allopathic medicine. In Figure 2, another folk healer is seen displaying his medicine in a market.

## CONCLUSION

Of *Sikkim*’s rich plant biodiversity, the 31 medicinal plants in the table 1 are the most used in traditional healing practices. Scientific validation, reverse pharmacological and observational studies are required for them, and for the various belief-based treatments of the three *Sikkim* communities. Then, this traditional knowledge could be utilized for primary health care, and to generate employment. *Sikkim*’s folk traditions are gradually declining in this Trans Himalayan region, as few in the new generation are coming forward to adopt folk healing practice as a profession. There is a significant shift in the socioeconomic pattern of so the folk healers of *Sikkim*, the department of AYUSH is actively trying to revitalize *Sikkim*’s local health traditions and folk healing practices by conducting training workshops and seminars. The challenges are to educate folk healers about their weaknesses and strengths, to attract young stars to adopt this profession by means of monetary benefits, and to preserve the knowledge and biodiversity. The NGOs working for traditional medicine are also trying to establish an association of folk healers of *Sikkim* for the preservation and promotion of their age old traditions. The traditional knowledge and the position of folk healing practices are not valued adequately in the face of modernization. The greatest challenge in this new millennium is to integrate the traditional knowledge with modern medicine, identify molecules for use in modern medicine, to decelerate the pace of environmental degradation, and to make the region’s economic development eco-friendly.
